# Effects of Ginkgo biloba in dementia: systematic review and meta-analysis

**DOI:** 10.1186/1471-2318-10-14

**Published:** 2010-03-17

**Authors:** Stefan Weinmann, Stephanie Roll, Christoph Schwarzbach, Christoph Vauth, Stefan N Willich

**Affiliations:** 1 Institute for Social Medicine, Epidemiology and Health Economics, Charité University Medicine, Luisenstrasse 57, 10117 Berlin, Germany; 2 Center for Health Economics and Health Systems Research, Gottfried Wilhelm Leibniz University, Königsworther Platz 1, 30167 Hannover, Germany

## Abstract

**Background:**

The benefit of Ginkgo biloba has been discussed controversially. The aim of this review was to assess the effects of Ginkgo biloba in Alzheimer's disease as well as vascular and mixed dementia covering a variety of outcome domains.

**Methods:**

We searched MEDLINE, EMBASE, the Cochrane databases, CINAHL and PsycINFO for controlled trials of ginkgo for Alzheimer's, vascular or mixed dementia. Studies had to be of a minimum of 12 weeks duration with at least ten participants per group. Clinical characteristics and outcomes were extracted. Meta-analysis results were expressed as risk ratios or standardized mean differences (SMD) in scores.

**Results:**

Nine trials using the standardized extract EGb761^® ^met our inclusion criteria. Trials were of 12 to 52 weeks duration and included 2372 patients in total. In the meta-analysis, the SMDs in change scores for cognition were in favor of ginkgo compared to placebo (-0.58, 95% confidence interval [CI] -1.14; -0.01, p = 0.04), but did not show a statistically significant difference from placebo for activities in daily living (ADLs) (SMD = -0.32, 95% CI -0.66; 0.03, p = 0.08). Heterogeneity among studies was high. For the Alzheimer subgroup, the SMDs for ADLs and cognition outcomes were larger than for the whole group of dementias with statistical superiority for ginkgo also for ADL outcomes (SMD = -0.44, 95% CI -0.77; -0.12, p = 0.008). Drop-out rates and side effects did not differ between ginkgo and placebo. No consistent results were available for quality of life and neuropsychiatric symptoms, possibly due to the heterogeneity of the study populations.

**Conclusions:**

Ginkgo biloba appears more effective than placebo. Effect sizes were moderate, while clinical relevance is, similar to other dementia drugs, difficult to determine.

## Background

The standardized Ginkgo biloba extract EGb 761^® ^is one of the most widely used herbal remedies for dementia and cognitive impairment and remains one of the best evaluated and characterized extracts [1]. Since 2000, according to the current ATC-classification, Ginkgo biloba special extract is listed in the group of anti-dementia drugs together with cholinesterase inhibitors and memantine. However, most efficacy and effectiveness studies are small, suffer from methodological limitations and are subject to considerable controversy.

Recently, the discussion about the benefits of Ginkgo biloba extracts for different indications has again been revitalized after the publication of two major trials. One study [2] with 176 participants could not demonstrate any evidence of effectiveness of 120 mg Ginkgo biloba in mild to moderate dementia. In addition, the Ginkgo Evaluation of Memory (GEM) study [3] found no favorable effects of 240 mg EGb 761^® ^for the prevention of dementia onset in older people without or with only mild cognitive impairment. Whereas the GEM trial was large enough to prove the robust result of a lacking effect of Ginkgo biloba in dementia prevention and overall mortality in people without dementia [4], the randomized controlled trial (RCT) of McCarney et al. has been criticized due to methodological problems and insufficient sample size [5,6].

Most previous reviews have shown inconsistent results and fail to draw firm conclusions whether Ginkgo biloba has patient-relevant benefits in people with a diagnosis of dementia [7,8]. In the meantime, new studies have been published. A major limitation of the available Cochrane Review on the effectiveness of Ginkgo biloba is the combined evaluation of cognitive decline and dementia. No subgroup analyses were performed [7]. As no valid definition of cognitive impairment has been used, and given the finding of differential effects of other anti-dementia drugs on dementia and cognitive impairment [9], it appears no longer reasonable to pool these two indications. Furthermore, the uncertainty regarding mild cognitive impairment (MCI) as a clinical entity raises the question as to the scientific validity of MCI trials. In addition, the German Health Technology Assessment Institute IQWiG (Institute for Quality and Efficiency in Health Care) published a favorable report on the effectiveness of Ginkgo biloba, which was, however, limited to Alzheimer's disease and contradicted the Cochrane review [10]. Taking into account the overlap between different types of dementia [11] and the limited significance of dementia subtyping in routine use of anti-dementia drugs, there is no clear evidence for substantial differences in the effectiveness of those drugs in vascular or Alzheimer's dementia. As most anti-dementia drugs including ginkgo are prescribed without comprehensive assessment of the dementia subtype, and given continuing high prescription rates of ginkgo to people with an established dementia diagnosis in some countries, we felt that further decision support is necessary for this kind of routine use of the substance. Therefore, we performed a systematic review on the effects of Ginkgo biloba in Alzheimer's disease as well as vascular and mixed dementia covering a variety of outcome domains.

## Methods

### Data sources

We used a sensitive search strategy based on Cochrane and other systematic reviews in the field. We searched the following databases: MEDLINE (January 1, 1966, to August 2008), EMBASE (January 1, 1980 to August 2008), PsycINFO (January 1, 1982, to August 2008), CINAHL (Cumulative Index to Nursing and Allied Health Literature), the Cochrane Database of Systematic Reviews, and the Cochrane Controlled Trials Register (until Issue 3, 2008). The following search terms were used for dementia (with * characterizing a wildcard, and the items AND and OR being used as boolean functions): dementia; (senile* AND dement*); Alzheimer*; ((cognit* OR memory* OR mental*) AND (decline OR impair* OR los* OR deteriorat* OR diminish* OR insufficien* OR degenerat*)). The following search terms were used for Ginkgo biloba: ginkgo; ginko; gingko; bilobalid*; tebonin; egb 761; li 1370. To identify clinical trials, we used the following search terms: (clinical AND trial*); random*; placebo*; (controlled AND trial*); (multicent* AND stud*); (comparative AND stud*); follow-up; (research AND design). All search terms within the indication (dementia), ginkgo biloba and clinical trials section were searched individually in each database and combined together using the "OR" boolean. The results of each of the three sections were then combined by utilizing the "AND" boolean. Furthermore, the reference sections of systematic and narrative reviews were screened for primary publications. In addition, the manufacturer of EGb 761^®^, Dr. Willmar Schwabe GmbH & Co. KG, Karlsruhe, Germany, was queried and information was requested as needed.

### Selection and study characteristics

We selected controlled clinical trials, with or without randomization, assessing the effects of treating people with a diagnosis of Alzheimer's disease, vascular or mixed dementia according to internationally valid diagnostic criteria, with a standardized Ginkgo biloba extract. Further study inclusion criteria were the use of an internationally accepted diagnostic for the dementia diagnosis such as the International Classification of Diseases (ICD) [12], the Diagnostic and Statistical Manual of Mental Disorders (DSM) [13], the NINCDS-ADRDA (National Institute of Neurological and Communicative Disorders and Stroke, Alzheimer's Disease and related Disorders Association) [14], or the NINDS-AIREN criteria (National Institute for Neurological Disorders and Stroke and Association Internationale pour la Recherche et l'Enseignement en Neurosciences) [15]; a minimum treatment duration of 12 weeks; a minimum number of participants of ten per group; and the availability of a full-text publication. Exclusion criteria were studies with a majority of people with specific types of non-vascular and non-Alzheimer's dementia, such as Lewy-body dementia or dementia due to Parkinson's disease; and a publication language other than English, German, French, Italian or Spanish.

### Data extraction and critical appraisal of studies

Study selection and appraisal of studies were performed independently by two researchers (SW and SR). Disagreement was resolved by consensus. Information was extracted via a standardized checklist included study design, duration of the study, comparability of study groups with respect to sex, age, baseline scores of cognitive, psychopathological and activities of daily living scales and clinical outcomes on different rating scales in the following domains: cognition, activities of daily living (ADLs), neuropsychiatric symptoms, patients' quality of life, response according to the studies' definitions and drop out rates due to side effects. The study and publication quality was assessed on the basis of whether the following quality criteria had been adequately fulfilled: randomization and allocation concealment, blinding of patients and investigators, sample size estimation, handling and reporting of study discontinuations, application of the intent-to-treat principle, relevant data inconsistencies, and report of study funding. All clinical outcomes were assessed in the intent-to-treat samples. An intent-to-treat analysis was accepted when a last-observation-carried forward analysis was performed, or when no drop-outs occurred and all patients were included in the evaluation.

The following rating scales were accepted for clinical outcomes: (1) cognition: Alzheimer's Disease Assessment Scale, cognitive subscale (ADAS-cog) [16], Syndrom-Kurztest (SKT) [17]; (2) ADLs: GERRI [18], Nürnberger Alters-Alltagsaktivitäten-Skala (NAA) and Nürnberger Alters-Beobachtungsskala (NAB) [19], GBS-ADL **(**Gottries-Bråne-Steen - Activities of daily living scale) [20], ADL-IS (Alzheimer's Disease Activities-of-Daily-Living International Scale) [21], (3) neuropsychiatric symptoms: Geriatric Depression Scale (GDS) [22], Hamilton Depression Rating Scale (HAMD) [23], Montgomery Asberg Depression Rating Scale (MADRS) [24] and Neuropsychiatric Inventory (NPI) [25]; and (4) quality of life: Quality of Life in Alzheimer's Disease (QOL-AD) [26], DEMQOL-Proxy (Quality of Life Questionnaire for people with dementia, rated by caregivers) [27], PDS (Progressive Deterioration Scale) [28]. In addition, we intended to pool the response rates with response definitions as used in the studies.

### Statistical analysis

If feasible and meaningful, data were pooled by means of meta-analysis. Effect measures presented in this publication were reported as Mantel Haenszel risk ratios (RRs) including 95% confidence intervals (CIs) for binary data. Effects on rating scales were expressed as standardized mean differences (SMDs) with the 95% CIs, N and p values. A random-effects model was used to calculate a pooled effect estimate, because we expected considerable heterogeneity. When different dose levels were used in different study arms, the study arm with the higher dosage was chosen, and a sensitivity analysis was performed. Further sensitivity analyses were performed for type of dementia (Alzheimer's dementia versus vascular or mixed dementia) and exclusion of studies with a high number of participants without dementia.

Statistical significance was assumed in case of p < 0.05. Heterogeneity of effect sizes was evaluated by the I^2 ^statistic. An alpha error p < 0.05 and/or I^2 ^of at least 50% were taken as indicators of substantial heterogeneity of outcomes. In this case, we examined the effect of removing single studies with special study designs on the results and on the heterogeneity. If meta-analyses were not possible, the results of individual studies are presented. Meta-analyses were performed using Review Manager, version 5 (The Cochrane Collaboration, Oxford, England) for all calculations.

### Role of funding source

Funding for this review was provided via an unrestricted grant from Dr. Willmar Schwabe GmbH & Co. KG, Karlsruhe, Germany.

## Results

### Study characteristics

**Figure 1:**
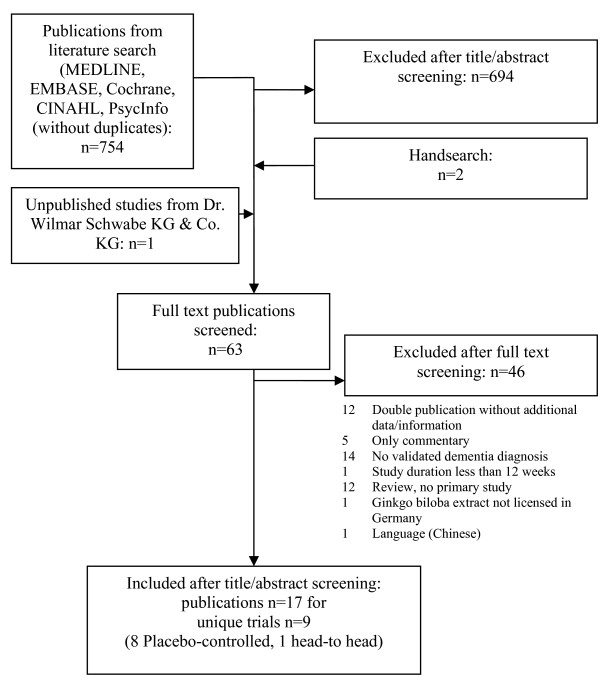
**Flow chart of study identification and selection**.

Our literature search in MEDLINE, EMBASE, Cochrane, PsycINFO and CINAHL yielded 754 clinical publications. An unpublished manuscript of one study (which has been published meanwhile) could be identified via the manufacturer request. Two further publications were added after hand searching journals and reference lists of papers, with one additional unpublished manuscript being supplied by the manufacturer. On the whole, 63 publications were included in the full-text screening. 17 publications reporting nine individual studies, all using the standardized extract EGb 761^®^, met our inclusion criteria (Figure 1). EGb 761^® ^is a dry extract from Ginkgo biloba leaves, extraction solvent: acetone 60% (w/w). The extract is adjusted to 22.0-27.0% ginkgo flavonoids, calculated as ginkgo flavone glycosides and 5.0-7.0% terpene lactones consisting of 2.8-3.4% ginkgolides A, B, C and 2.6-3.2% bilobalid. It contains less than 5 ppm ginkgolic acids. There was no study with other ginkgo product that met our inclusion criteria.

All included studies were randomized. The randomization procedure was rated as sufficient in all cases. However, allocation concealment remained unclear in three studies (table 1). All studies were described as double-blind, although in four studies, it was not explicitly stated that the outcome assessment was blinded. Eight studies were placebo-controlled [[2,29-34], Ihl R, Bachinskaya N, Korczyn AD, Tribanek M, Hoerr R, Napryeyenko O: Efficacy and Safety of a Once-Daily Formulation of Ginkgo biloba Extract EGb 761^® ^in Dementia with Neuropsychiatric Features. A Randomized Controlled Trial, submitted], and one study was a head-to-head trial with donepezil as comparison group [35]. Overall, the methodological quality of the included studies was moderate to good, with most studies using an intent-to-treat analysis which was judged as adequate. Two studies were performed in Germany, two in the USA, and two in Ukraine, whereas one study was performed in Bulgaria, the Netherlands and Great Britain, respectively. In total, 2372 patients were included and treated. All studies included patients with Alzheimer's disease. Six studies included also patients with vascular dementia [[29,30], Ihl R, Bachinskaya N, Korczyn AD, Tribanek M, Hoerr R, Napryeyenko O: Efficacy and Safety of a Once-Daily Formulation of Ginkgo biloba Extract EGb 761^® ^in Dementia with Neuropsychiatric Features. A Randomized Controlled Trial, submitted] or vascular dementia plus mixed dementia [[2,32,34], Ihl R, Bachinskaya N, Korczyn AD, Tribanek M, Hoerr R, Napryeyenko O: Efficacy and Safety of a Once-Daily Formulation of Ginkgo biloba Extract EGb 761^® ^in Dementia with Neuropsychiatric Features. A Randomized Controlled Trial, submitted]. One study included a majority of participants with age-associated memory impairment. This study was subjected to sensitivity analysis [34]. All studies included only patients with mild or moderate dementia (table 2). Some studies did not include patients with substantial neuropsychiatric or behavioral symptoms [30,31,33,34,36], whereas in two studies, the presence of depression or behavioral symptoms of moderate intensity was an explicit inclusion criterion [[32], Ihl R, Bachinskaya N, Korczyn AD, Tribanek M, Hoerr R, Napryeyenko O: Efficacy and Safety of a Once-Daily Formulation of Ginkgo biloba Extract EGb 761^® ^in Dementia with Neuropsychiatric Features. A Randomized Controlled Trial, submitted]. Patients with severe or uncontrolled somatic disorders were excluded from all trials except one [2].

**Table 1:** Methodological quality of included studies

Study Authors, date	Randomization/allocation concealment	Blinding of patients/outcomes assessment	Prior estimate of sample size	Withdrawals per group reported	ITT analysis adequate	Report of measures of precision	Data inconsistencies	Funding reported
Kanowski et al. 1996/2003 [29,36]	Yes/yes	Yes/yes	Partly^a^	Partly^b^	Yes	Yes	No	No

Le Bars et al. 1997/2000 [30,37]	Yes/yes	Yes/unclear	Yes	Yes	Yes	Yes	No	Yes

Maurer et al. 1997 [31]	Yes/unclear	Yes/unclear	No	Yes	No	No	No	No

van Dongen et al. 2000 [34,45]	Yes/yes	Yes/yes	Yes	Yes	No	Yes	Yes^c^	Yes

Schneider et al. 2005 [33]	Yes/yes	Yes/yes	Yes	Yes	Yes	Yes	No	Yes

Yancheva et al 2009 [35]	Yes/unclear	Yes/unclear	No	Yes	Yes	Yes	Yes	Yes

Napryeyenko et al. 2007 [32]	Yes/unclear	Yes/unclear	Yes	Yes	Yes	Yes	No	Yes

McCarney et al. 2008 [2]	Yes/yes	Yes/yes	Partly^a^	Yes	Yes	Yes	No	Yes

Ihl et al. 2009	Yes/yes	Yes/yes	No	Yes	Yes	Yes	No	Yes

a: Less patients were included than planned; b: Drop-out rates were reported, but without reference to study arm; c: Multiple re-randomization schemes.

ITT = Intent-to-treat analysis.

**Table 2:** Characteristics of included studies and participants, Ginkgo biloba for dementia

Study Authors, date	Inclusion criteria	Setting	Duration (weeks)	Treatment groups	**N**^a^	Drop out rate N (%)	Age **Mean (SD)**^b^	Sex % Female	Baseline cognition	
									Scale (SD)	Mean
Kanowski et al. 1996/2003 [29,36] *All patients*	DSM-III-R Alzheimer's or vascular dementia, age > 54 ys, SKT 6-18, MMSE 13-25, no cerebral atrophy, MADRS < 41	Outpatients	24	Ginkgo biloba 240 mg Placebo	106 99	27 (25) 22 (22)	72 (10) 72 (10)	68 71	SKT	10,5 (3,2) 11,2 (3,3)
							
*Only Alzheimer*					79 79	n.a.	72 (10) 72 (10)	71 73		10,3 (3,1) 10,9 (3,3)

Le Bars et al. 1997/2000 [30,37] *All patients*	DSM-III-R and ICD-10 Alzheimer's or vascular dementia, age > 44 ys, MMSE 9-26, GDS 3-6, no psychiatric comorbidity	Outpatients	52	Ginkgo biloba 120 mg Placebo	155 154	77 (50) 95 (62)	69 (47-89) 69 (45-90)	51 56	ADAS-cog	20,0 (16,0)^c^ 20,5 (14,7)^c^
							
*Only Alzheimer*					120 116	n.a.	68 (47-89) 68 (45-90)	54 62		19,7 (16,4) 20,2 (15,2)

Maurer et al. 1997 [31]	DSM-III-R and NINCDS/ADRDA Alzheimer's or vascular dementia, age 50-80 ys, BCRS 3-5, Hachinski-Ischemia Score ≤ 4, no psychiatric comorbidity.	Outpatients	12	Ginkgo biloba 240 mg Placebo	9 9	1 (10) 1 (10)	68,5 (6,0) 60,6 (8,9)	56 44	ADAS-cog	31,2 (12,6) 36,1 (15,2)

van Dongen et al. 2000 [34,45]	DSM-III-R or ICD-10 Alzheimer's or vascular dementia, or AAMI (clinical diagnosis), age > 49, SKT 8-23, AAMI: MAC-Q ≥ 12 and no dementia (SIDAM), GDS < 11, IQ > 80, no psychiatric comorbidity	Nursing home/old age home	24	Ginkgo biloba 160 mg or 240 mg Placebo	79 44	14 (18) 4 (9)	83 (n.a.) 83 (n.a.)	86 82	MMSE	18,0 (4,9) 18,7 (4,6)

Schneider et al. 2005 [33]	NINCDS/ADRDA probable Alzheimer dementia, age ≥ 60, MMSE 10-24, Hachinski-Ischemia Score ≤ 4, HAM-D < 16, no psychiatric comorbidity	Outpatients	26	Ginkgo biloba 240 mg Ginkgo biloba 120 mg Placebo	170 169 174	30 (18) 34 (20) 39 (22)	78 (7) 79 (7) 78 (7)	56 50 52	ADAS-cog	24,8 (12,7) 24,7 (11,9) 25,0 (11,1)

Yancheva et al 2009 [35]	NINCDS/ADRDA probable Alzheimer dementia, age ≥ 50, Clock-drawing Test Score < 6, SKT 9-23, NPI ≥ 5	Outpatients	22	Ginkgo biloba 240 mg Donepezil 10 mg Combination of both	31 32 31	1 (3) 4 (1) 2 (6)	69 (8) 66 (8) 68 (9)	55 84 68	SKT	15,7 (4,7) 17,4 (4,2) 17,4 (4,4)

Napryeyenko et al. 2007 [32,51] *All patients*	NINCDS/ADRDA probable Alzheimer or probable NINDS/AIREN vascular dementia or mixed form, age ≥ 50, Clock-drawing Test Score < 6, SKT 9-23, HAMD17 < 20, NPI ≥ 3, TE4D ≤ 35	Outpatients	22	Ginkgo biloba 240 mg Placebo	198 197	4 (2) 5 (3)	65 (8) 63 (8)	86 82	SKT	15,6 (3,9) 15,4 (3,7)
							
*Only Alzheimer*					104 110	n.a.	66 (8) 64 (8)	67 71		16,4 (3,8) 15,8 (3,8)

McCarney et al. 2008 [2]	DSM-IV dementia, age ≥ 55, MMSE 12-26, presence of a caregiver, Ginkgo biloba was allowed until 2 weeks and cholinesterase inhibitors until 2 months before inclusion	Outpatients	24	Ginkgo biloba 120 mg Placebo	88 88	25 (28) 20 (23)	79,3 (7,8) 79,7 (7,5)	58 64	ADAS-cog	20,4 (8,2) 25,0 (10,3)

Ihl et al. 2009 *All patients*	NINCDS/ADRDA probable Alzheimer or probable NINDS/AIREN vascular dementia or mixed form, age ≥ 50, Clock-drawing Test Score < 6, SKT 9-23, HAMD17 < 20, NPI ≥ 3, TE4D ≤ 35	Outpatients	24	Ginkgo biloba 240 mg Placebo	202 202	16 (8) 12 (6)	65 (10) 65 (9)	69 66	SKT	16,7 (3,9) 17,2 (3,7)
							
*Only Alzheimer*					163 170		65 (10) 64 (9)	67 65		16,4 (3,8) 17,0 (3,8)

a: N includes all patients randomized that completed at least one post-treatment evaluation; b: SD = standard deviation (one number) or range (two numbers).

ADAS-cog = Alzheimer's Disease Assessment Scale cognitive subscale (0-70); AAMI = age-associated memory impairment; BCRS = Brief Cognitive Rating Scale; GDS = Global Deterioration Scale; MAC-Q = Memory Assessment Clinical Questionnaire; MMSE = Mini Mental State Examination (0-30); n.a. = not available; SIDAM = Structured Interview for the Diagnosis of Dementia of the Alzheimer Type; SKT = Syndrom Kurz-Test (0-27), TE4D = Test for Early Detection of Dementia with Discrimination from Depression (ranges of the scale score in brackets, the underlined numbers indicate highest symptom load or disturbance).

The 12-26 weeks outcomes of the studies for the whole patient group together (Alzheimer's disease, vascular dementia or mixed dementia) are presented in the next section. We present the results within different outcome domains. Furthermore, results are compared with the 52-weeks outcomes of the only long-term study [30]. Within the respective sections, we present data from the subgroup of patients with Alzheimer's disease. Analyses of other subgroups are reported separately. For all outcomes, negative change scores indicate an improvement.

### Clinical outcomes

#### Cognition

**Figure 2:**
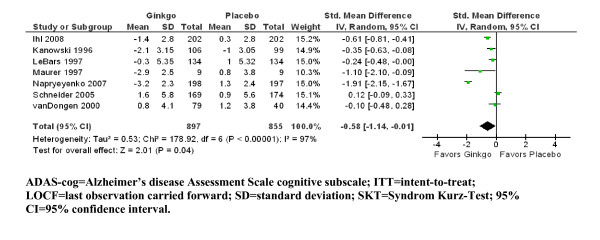
**ITT/LOCF change scores for cognition outcomes (ADAS-cog, SKT) by individual trial and pooled standardized mean difference compared with placebo**.

In all included studies (n = 9), cognition was evaluated. Three studies used the ADAS-cog [2,30,33], whereas in five studies [[32,34-36], Ihl R, Bachinskaya N, Korczyn AD, Tribanek M, Hoerr R, Napryeyenko O: Efficacy and Safety of a Once-Daily Formulation of Ginkgo biloba Extract EGb 761^® ^in Dementia with Neuropsychiatric Features. A Randomized Controlled Trial, submitted], the SKT and in one study [31], both outcomes scales were used. Due to the superior sensitivity of the SKT in mild to moderate dementia, we used the SKT for the latter study. Change scores for ADAS-cog ranged from -0.3 to 1.3 in the ginkgo groups and from 0.9 to 1.0 in the placebo groups, whereas change scores for SKT ranged from -3.2 to -0.8 in the ginkgo groups and from -1.2 to 1.3 in the placebo groups. Standardized change scores were greater for ginkgo than for placebo, with SMD = -0.58 (95% CI -1.14; -0.01, z = 2.01, N = 7, p = 0.04) (Figure 2). However, heterogeneity was substantial (χ^2 ^= 178.92, I^2 ^= 97%).

Six studies with separate analyses for patients with Alzheimer's disease could be included for the subgroup evaluation. For the Alzheimer subgroup, the standardized change scores (ADAS-cog or SKT) were greater for ginkgo than for placebo, with SMD = -0.63 (95% CI -1.16; -0.10, z = 2.35, N = 6, p = 0.02). Heterogeneity remained high also in this subgroup (χ^2 ^= 95.96, I^2 ^= 95%).

#### Activities of daily living (ADLs)

**Figure 3:**
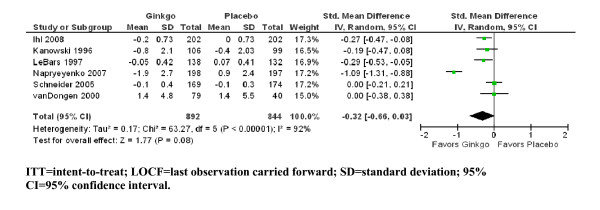
**ITT/LOCF change scores for activities of daily living outcomes by individual trial and pooled standardized mean difference compared with placebo**.

In eight of the nine included studies, ADLs were evaluated. Three studies [2,30,33] used the GERRI, one study used the Nürnberger Alters-Alltagsaktivitätenskala (NAA, self-assessed) [34], one trial used the Nürnberger Alters-Beobachtungsskala (NAB, caregiver rated) [36], one study used the ADL-IS (Ihl R, Bachinskaya N, Korczyn AD, Tribanek M, Hoerr R, Napryeyenko O: Efficacy and Safety of a Once-Daily Formulation of Ginkgo biloba Extract EGb 761^® ^in Dementia with Neuropsychiatric Features. A Randomized Controlled Trial, submitted), and two studies used the GBS-ADL subscale [32,35]. Change scores ranged from -0.1 to -0.05 in the ginkgo groups and from -0.1 to 0.08 in the placebo groups for GERRI, from -1.9 to -0.8 in the ginkgo groups and 0.8 to 0.9 in the placebo groups for GBS-ADL, from -0.2 to -0.16 in the ginkgo groups and 0 in the placebo groups for ADL-IS, and from -1.0 to -0.8 in the ginkgo groups and -0.4 in the placebo groups for NAB. The change score was 1.4 in both the placebo group and the ginkgo group in the study where the NAA was used as outcome measure. There were no statistically significant differences in ADL change scores between ginkgo and placebo, with SMD = -0.32 (95% CI -0.66; 0.03, z = 1.77, N = 6, p = 0.08) (Figure 3). Again, heterogeneity was substantial (χ^2 ^= 83.27, I^2 ^= 92%).

For the Alzheimer subgroup (n = 5 trials) the standardized change scores for ADL outcomes differed from the whole group of dementia. In patients with Alzheimer's disease, there was a statistical superiority for Ginkgo biloba compared to placebo (SMD = -0.44, 95% CI -0.77; -0.12, z = 2.67, p = 0.008). Heterogeneity was only slightly lower in this subgroup (χ^2 ^= 32.34, I^2 ^= 88%).

#### Neuropsychiatric and behavioral symptoms

Neuropsychiatric symptoms were assessed in seven trials (n = 4 NPI, n = 3 HAMD, n = 1 GDS and n = 1 MADRS). In some studies, depression scales were used to exclude patients with clinically relevant depression. In these studies, the investigators wanted to make clear that effects on cognitive parameters could not be explained by antidepressive effects. In two trials, which included dementia patients with pre-existing neuropsychiatric symptoms, there was a superiority of ginkgo compared to placebo. Change scores ranged from -6.5 to -3.2 in the ginkgo groups and from 2.4 to 0 in the placebo groups for NPI [[32], Ihl R, Bachinskaya N, Korczyn AD, Tribanek M, Hoerr R, Napryeyenko O: Efficacy and Safety of a Once-Daily Formulation of Ginkgo biloba Extract EGb 761^® ^in Dementia with Neuropsychiatric Features. A Randomized Controlled Trial, submitted]. These differences were statistically significant in both studies. However, four studies with non-depressed and non-behaviorally disturbed patients did not show any difference between ginkgo and placebo [2,33,34,36] for neuropsychiatric and behavioral symptoms. Results for the Alzheimer subgroup did not differ significantly from the whole dementia group.

#### Quality of life

Quality of life was assessed in three studies. In one study, the QOL-AD scale was used as primary outcome parameter [2]. Whereas two studies showed no difference between ginkgo and placebo in the 24-26 weeks evaluation for PDS [33] and QOL-AD [2], one study (Ihl R, Bachinskaya N, Korczyn AD, Tribanek M, Hoerr R, Napryeyenko O: Efficacy and Safety of a Once-Daily Formulation of Ginkgo biloba Extract EGb 761^® ^in Dementia with Neuropsychiatric Features. A Randomized Controlled Trial, submitted) yielded a statistically significant superiority of ginkgo in the improvement of quality of life compared to placebo. There was an improvement of 3.4 points in the DEMQOL-proxy scale for the ginkgo group compared to a 1.4 improvement in the placebo group.

#### Side effects

Discontinuation rates due to side effects varied from 1% to 6% in the ginkgo groups and from 0 to 8% in the placebo groups. We could not find any differences between the pooled groups in side effects and discontinuation due to side effects.

#### Sensitivity analysis

Response was defined as an improvement of SKT or ADAS-cog sores by ≥ 4 points [36,37], improvement of SKT by at least 3 points [[32,34], Ihl R, Bachinskaya N, Korczyn AD, Tribanek M, Hoerr R, Napryeyenko O: Efficacy and Safety of a Once-Daily Formulation of Ginkgo biloba Extract EGb 761^® ^in Dementia with Neuropsychiatric Features. A Randomized Controlled Trial, submitted], improvement in GERRI [30], NPI improvement of ≥ 4 points [[2], Ihl R, Bachinskaya N, Korczyn AD, Tribanek M, Hoerr R, Napryeyenko O: Efficacy and Safety of a Once-Daily Formulation of Ginkgo biloba Extract EGb 761^® ^in Dementia with Neuropsychiatric Features. A Randomized Controlled Trial, submitted], much or very much improvement in the Clinical Global Impression Scale (CGI) [31,36], or an improvement or no change in the Clinical Global Impression of Change Scale (CGIC) [33]. These variations in the response definitions limit the validity of this outcome measure, and we decided not to evaluate response rates.

In one study [2], participants were allowed to continue a stable intake of cholinesterase inhibitors. This led to one third of participants being prescribed such substances. Furthermore, ginkgo was allowed to be taken until two weeks before the baseline evaluation (cholinesterase inhibitors intake: placebo 10%; ginkgo 25%) thus contributing to a possible spill-over effect into the treatment phase. For most outcome parameters there were no post-baseline-scores but only adjusted differences in means available. Therefore, this study was not included into meta-analyses. Removing one further trial with only 30% of patients with a diagnosis of dementia [34] did not significantly change our results. After excluding this study, the SMD for cognition was -0.66 (95% CI -1.29; -0.03, z = 2.06, N = 6, p = 0.04), the SMD for ADLs was -0.37 (95% CI -0.76; 0.01, z = 1.88, N = 5, p = 0.06), and the RR for response was 1.85 (95% CI 1.17; 2.56, z = 2.93, N = 6, p = 0.009) for the group of all dementias. In all cases, heterogeneity remained high (I^2 ^above 80%).

A funnel plot of standard errors of log RRs (for response) against log RRs showed a symmetrical distribution with no evidence of publication bias. In the high dosage group (240 mg Ginkgo biloba), the effect was higher than in the low-dosage group (120 mg Ginkgo biloba) in all outcome domains. For 120 mg Ginkgo biloba, only one study [37] showed an advantage in cognition and ADLs compared to placebo.

In the only head-to-head trial with 96 participants, there was no difference after 22 weeks between Ginkgo biloba 240 mg, donepezil 10 mg and the combination treatment in any outcome parameter (SKT, NPI, GBS-ADL, HAMD and neuropsychological tests).

## Discussion

This review was performed because the efficacy and effectiveness of Ginkgo biloba in dementia treatment has been an issue of controversy against a background of an increasing individual and societal burden due to these disorders, and given only moderate effects of cholinesterase inhibitors and memantine [38,39]. With only symptomatic treatments available, the clinical relevance of all anti-dementia drugs would be to improve cognitive and neuropsychological symptoms and activities of daily living and/or to delay deterioration.

We found a statistically significant advantage of Ginkgo biloba compared to placebo in improving cognition for the whole group of patients with Alzheimer's disease, vascular or mixed dementia. Regarding activities of daily living, there was no significant difference for the whole dementia group. However, in the subgroup of patients with Alzheimer's disease, the advantage of Ginkgo biloba compared to placebo was statistically significant. The benefit of Ginkgo biloba in overall response rates was quite robust when all response definitions of the studies were accepted. However, the available response definitions differed considerably. Therefore, we did not pool the results.

There was no consistent evidence of a benefit of Ginkgo biloba in the treatment of neuropsychiatric symptoms. However, the presence of psychological or behavioral symptoms in dementia may be an effect modifier. Two studies that included patients with neuropsychiatric features yielded a clinically relevant effect in this outcome domain. Based on our results, no solid conclusions can be given for quality of life. Subgroup analyses showed that a dosage of 240 mg of ginkgo might be necessary to yield clinically relevant effects.

According to the evaluated studies, Ginkgo biloba extract seems to be well tolerated with rates of adverse effects and study withdrawals not being different between medication and placebo. Nevertheless, it has to be taken into account that randomized controlled trials are not suitable to evaluate rare events and medication interactions. The included studies were undertaken with the standardized extract EGb 761^®^. However, other ginkgo extracts and ginkgo-containing products may be associated with different side effects. During intake of EGb 761^®^, mild gastro-intestinal symptoms, headache, dizziness or allergic skin reactions have occasionally been reported. Single cases of bleeding, e.g. intracranial haemorrhage, have been reported in the context of an intake of Ginkgo biloba preparations. However, some of these products concerned were of unknown origin and quality, some were multi-ingredient products, and in most instances anti-platelet agents or anticoagulants were taken in addition. A review of controlled studies indicated that the ginkgo extract EGb 761^® ^does neither influence blood clotting nor bleeding time nor significantly potentiates the effects of anticoagulant or anti-platelet drugs [40]. In addition, a systematic review of case reports concluded that the clinical evidence for Ginkgo biloba causing bleeding is far from compelling [41,42].

We included efficacy as well as effectiveness studies in a variety of settings and regions where different additional psychosocial treatments were offered. In addition, in recent years the availability of cholinesterase inhibitors and memory clinics may have influenced the way dementia is seen and treated. With cholinesterase inhibitors being established treatments, it has become more difficult to run placebo- or actively controlled ginkgo trials in Western countries. The larger effect sizes in the Eastern European studies [[32], Ihl R, Bachinskaya N, Korczyn AD, Tribanek M, Hoerr R, Napryeyenko O: Efficacy and Safety of a Once-Daily Formulation of Ginkgo biloba Extract EGb 761^® ^in Dementia with Neuropsychiatric Features. A Randomized Controlled Trial, submitted] may indicate a higher differential efficacy of Ginkgo biloba compared to placebo in a setting with less specialized dementia care and less availability or reimbursement of anti-dementia drugs. These two studies were the ones with the highest effect sizes for cognition and activities of daily living outcomes and had extremely low drop-out rates. Therefore, the higher effect sizes in these studies might be a consequence of lower drop-out rates and less influence of statistical assumptions necessary to calculate last-observation-carried-forward analyses. In addition, treatment can only work optimally in compliant patients staying in the study for the whole follow-up period. When more people drop out of the treatment arm, this would lead to an underestimation of the effect size of the intervention.

In a situation where the clinical significance of the effects of anti-dementia drugs is increasingly questioned, and neither empirical data nor a consensus on minimally clinically important differences in outcome parameters is available [43], it is difficult to assess if the moderate effects of ginkgo on cognition and ADLs make a difference for the patients in the long run. The validity and reliability of clinical endpoints remain unclear with some early trials using outcomes that would not be accepted for modern studies. Furthermore, there was only one study with a 52-week-follow-up which showed a small but statistically significant advantage in cognition and daily activities. The effects did not differ in magnitude from the 26-week results. On the other hand, even a short follow-up time of 12 weeks was sufficient to separate ginkgo from placebo in one study [31]. This indicates that the duration of the study and the setting as well as methodological factors may be stronger outcome modifiers than the effects of the medication itself.

With Ginkgo biloba being prescribed for a variety of indications, external validity and generalisability of study results to the target population is of utmost importance. Using criteria for external validity assessment as proposed by Bornhöft et al. [44], we realized that none of the studies considered all those criteria. Some studies focused on the efficacy of ginkgo and tried to assure high internal validity with low selection, performance, detection and attrition bias [[33], Ihl R, Bachinskaya N, Korczyn AD, Tribanek M, Hoerr R, Napryeyenko O: Efficacy and Safety of a Once-Daily Formulation of Ginkgo biloba Extract EGb 761^® ^in Dementia with Neuropsychiatric Features. A Randomized Controlled Trial, submitted]. In these studies, patients with somatic or psychiatric comorbidity were excluded, and other medications were not allowed. This may limit the generalisability of the study results, although in most of the studies included in this review, the setting reflected the everyday conditions with most patients being treated by outpatient clinics or practice-based physicians. One study was performed only with persons living at old people's homes in the Netherlands and had an over-representation of the very old [34,45]. This may have contributed to the lack of ginkgo effectiveness versus placebo. Other studies were community-based pragmatic randomized trials with substantial clinical relevance. For example, in the McCarney et al. study [2], patients were included by general practitioners. The very pragmatic design, together with insufficient statistical power, however, may limit internal validity as many of the included patients were treated concurrently with cholinesterase inhibitors, which were allowed to be continued in both study arms. This may have led to a ceiling effect and the dilution of a significant effect [5]. A high external validity may therefore compromise internal validity, particularly in those cases where study designs reflect everyday conditions but fail to control other influences on the outcomes. As Bornhöft et al. note [46], in most ginkgo studies external validity has not sufficiently been addressed. With evidence of a considerable influence of contextual factors also in dementia treatment, it is probable that trial participation itself improves outcomes [47] and modifies the effects of medication. Therefore, it seems important to keep study conditions as close to the clinical reality.

Our results are consistent with the Cochrane data indicating a small advantage for ginkgo compared with placebo at 12 and 24 weeks in dementia [7]. However, we did not include trials where patients did not receive a validated dementia diagnosis. Due to our inclusion criteria, the overall methodological quality of studies was higher than in the Cochrane Review, which included some older studies without validated dementia diagnoses, less rigorous randomization and allocation schemes and, consequently, a higher risk of bias. In addition, we identified and included three recently performed trials. Two of these trials showed a considerable superiority of ginkgo in mild to moderate dementia. This may have contributed to the differences between ours and previous reviews. Contrary to previous reviews, we pooled the values of different outcomes scales of the same domain for meta-analysis (e.g. ADAS-cog and SKT for cognition). Given the paucity of studies using the same outcome scales, we felt that this was justified in order to increase the power of the meta-analysis.

Although there is some evidence of a Ginkgo biloba mode of action through mitochondrial stabilization and improvement of cerebral energy mechanism [48,49] in addition to hemodynamic effects, there is no consistent picture how this substance may alleviate dementia symptoms. However, this is also true for cholinesterase inhibitors.

Despite a considerable heterogeneity not fully explained by dementia type or ginkgo dosage, we think that ginkgo may be of benefit for a certain but unknown proportion of dementia patients. Against this background and in analogy with dementia prevention [3,50], it may be advisable to run a major multicenter study for dementia treatment to definitively answer the question of ginkgo effectiveness for different dementia subgroups in advanced health care systems. Treatment and setting should reflect everyday conditions as much as possible without compromising internal validity. We think that the hitherto gained results justify both symptomatic treatment of dementia and further research, as differential effects for Alzheimer's and vascular dementia are obvious and ginkgo is carried on being used as a complementary therapy in these disorders.

## Conclusion

We found a statistically significant advantage of Ginkgo biloba compared to placebo in improving cognition for the whole group of patients with Alzheimer's disease, vascular or mixed dementia. Regarding activities of daily living, there was no significant difference for the whole group. However, in the subgroup of patients with Alzheimer's disease, there was a statistically significant advantage of Ginkgo biloba compared to placebo. In a situation, where the clinical significance of the moderate effects of cholinesterase inhibitors and memantine as symptomatic treatments is increasingly been questioned, ginkgo biloba may not be an inferior treatment option for a considerable number of people with mild or moderate dementia. However, direct comparisons are lacking. A major multicenter study to compare the relative effectiveness of Ginkgo biloba and cholinesterase inhibitors for different dementia subgroups appears justified.

## Competing interests

The authors declare that they have no competing interests.

## Authors' contributions

SW, CV and SNW were primarily involved in conception and design of the study. SW, CS and SR were responsible for acquisition of data, analysis and interpretation of data, SW, SR, CS have been involved in drafting the manuscript, all authors had revised it critically and gave final approval of the version to be published.

## Pre-publication history

The pre-publication history for this paper can be accessed here:

http://www.biomedcentral.com/1471-2318/10/14/prepub
